# Evaluation of the efficacy and safety of conventional and biportal endoscopic decompressive laminectomy in patients with lumbar spinal stenosis (ENDO-B trial): a protocol for a prospective, randomized, assessor-blind, multicenter trial

**DOI:** 10.1186/s12891-021-04959-2

**Published:** 2021-12-20

**Authors:** Hyun-Jin Park, Sang-Min Park, Kwang-Sup Song, Ho-Joong Kim, Si-Young Park, Taewook Kang, Min-Seok Kang, Dong-Hwa Heo, Choon-Keun Park, Dong-Geun Lee, Jin-Sub Hwang, Jae-Won Jang, Jun-Young Kim, Jin-Sung Kim, Hong-Jae Lee, Joon-Hyeok Yoon, Chang-Won Park, Ki-Han You

**Affiliations:** 1 Department of Orthopedic Surgery, Spine Center, Kangnam Sacred Heart Hospital, Hallym University College of Medicine, 1, Singil-ro, Yeongdeungpo-gu, Seoul, 07441 South Korea; 2 Spine Center and Department of Orthopaedic Surgery, Seoul National University College of Medicine and Seoul National University Bundang Hospital, Seongnam, South Korea; 3 Department of Orthopaedic Surgery, Chung-Ang University, College of Medicine, Seoul, South Korea; 4 Department of Orthopaedics, Korea University College of Medicine, Anam Hospital, Seoul, South Korea; 5 Department of Orthopedic Surgery, Endoscopic Spine Surgery Center, Bumin Hospital, Seoul, South Korea; 6 Department of Neurosurgery, Endoscopic Spine Surgery Center, Seoul Bumin Hospital, Seoul, South Korea; 7 Department of Neurosurgery, The Leon Wiltse Memorial Hospital, Suwon, South Korea; 8 Department of Neurosurgery, Seoul St. Mary’s Hospital, The Catholic University of Korea, Seoul, South Korea; 9 Department of Neurosurgery, Daejeon St. Mary’s Hospital, The Catholic University of Korea, Seoul, South Korea

**Keywords:** Lumbar spinal stenosis, Biportal endoscopic spine surgery, Decompressive laminectomy

## Abstract

**Background:**

Recent studies on biportal endoscopic spine surgery in patients with lumbar spinal stenosis have reported good clinical results. However, these studies have been limited by the small sample sizes and use of a retrospective study design. Therefore, we aim to compare the efficacy and safety of biportal endoscopic decompressive laminectomy with those of conventional decompressive laminectomy in a multicenter, prospective, randomized controlled trial.

**Methods:**

This study will include 120 patients (60 per group, aged 20–80 years) with 1- or 2-level lumbar spinal stenosis, who will be recruited from six hospitals. The study will be conducted from July 2021 to December 2024. The primary outcome (Oswestry Disability Index at 12 months after surgery) will be evaluated through a modified intention-to-treat method. The secondary outcomes will include the following: visual analog scale score for low back and lower extremity radiating pain, EuroQol 5-dimensions score, surgery satisfaction, walking time, postoperative return to daily life period, postoperative surgical scars, and some surgery-related variables. Radiographic outcomes will be analyzed using magnetic resonance imaging or computed tomography. All outcomes will be evaluated before the surgery and at 2 weeks, 3 months, 6 months, and 12 months postoperatively. This protocol adheres to the Standard Protocol Items: Recommendations for Interventional Trials (SPIRIT) guidelines for reporting of clinical trial protocols.

**Discussion:**

It is hypothesized that the efficacy and safety of biportal endoscopic and conventional decompressive laminectomy will be comparable in patients with lumbar spinal stenosis. The results of this trial will provide a high level of evidence for the efficacy and safety of the biportal endoscopic technique in patients with lumbar spinal stenosis and facilitate the development of clinical practice guidelines. Furthermore, the results of this study may indicate the feasibility of the biportal endoscopic technique for other types of spinal surgery.

**Trial registration:**

The ENDO-B trial is registered at Clinical Research Information Service (CRIS, cris.nih.go.kr) (KCT0006057; April 52,021).

## Background

Lumbar spinal stenosis (LSS) is a disease that causes several symptoms, such as low back pain and intermittent claudication, due to the narrowing of the central spinal canal, nerve root canal, or intervertebral space [1]. With an aging demographic, surgical treatment for LSS has become increasingly common. Among the various surgical approaches for this condition, decompressive laminectomy is the most frequently used method [2]. However, this conventional method has been associated with complications such as intraoperative bleeding, postoperative pain, instability, and damage of the paraspinal muscles [3]. An emerging alternative method is minimally invasive surgery, which preserves the existing anatomical structures. The most frequently used minimally invasive surgery is unilateral laminectomy bilateral decompression (ULBD) [4, 5]. Compared with conventional laminectomy, ULBD showed comparable clinical outcomes, and it was superior in terms of postoperative recovery time, time to mobilization, and opioid use [2, 6].

In 1973, Parvis Kambin introduced the concept of a transforaminal approach, which involves the percutaneous placement of Craig’s cannula. In 1991, he performed the first endoscopic spine surgery using a cannula for interlaminar and transforaminal endoscopy [7]. Endoscope-assisted spinal surgery has several advantages, such as less approach-related trauma, preservation of the epidural blood supply, and reduced epidural scarring and fibrosis compared to those performed with conventional methods [8]. The biportal endoscopic spine surgery, first introduced by Soliman in 2013, is a technique that permits the free movement of instruments through the two portals [9]. It has a good field of view, as the camera can be placed at various angles; this facilitates safe and sufficient decompression. Furthermore, the equipment used for the knee and shoulder arthroscope can be used for the spine without the need for additional changes in settings. Moreover, it allows the use of the same instruments as in conventional spine surgery. Since the biportal endoscopic technique only requires small incisions of less than 1 cm, damage to the surrounding normal tissues is minimized; this results in fewer complications, such as pain and adhesion, after surgery.

Previous studies evaluating the use of the biportal endoscopic technique in LSS surgery have reported good clinical results; however, the strength of evidence has been limited by the use of a retrospective study design and small sample sizes [10, 11]. There is currently a lack of level 1 studies to establish the efficacy and stability of the biportal endoscopic technique in comparison with conventional methods. Therefore, we aim to compare the efficacy and safety of biportal endoscopic decompressive laminectomy with those of conventional decompressive laminectomy in a multicenter, prospective randomized controlled trial (RCT). Our hypothesis is that the efficacy and safety of these two methods are similar in patients with LSS.

## Methods/design

### Study design and inclusion criteria

The design and protocol of this multicenter, assessor-blind, prospective RCT were approved by the institutional review board of the participating hospitals from Korea. From July 2021 to December 2024, a total of 120 adults (aged 20–80 years) with radiating pain in the lower extremities or neurogenic claudication will be recruited from six hospitals. Patients will be included if they 1) have decided to undergo 1- or 2-level decompressive laminectomy for lumbar central canal stenosis, 2) are able to provide informed consent, and 3) are willing to participate and comply with our proposed follow-up protocol. The exclusion criteria were as follows: 1) spondylolisthesis (Meyer grade ≥ II), 2) a history of lumbar spinal surgery at the same level, 3) degenerative lumbar scoliosis (Cobb angle > 20°), 4) stenosis not caused by degeneration or intervertebral disc herniation, 5) other spinal diseases (e.g., ankylosing spondylitis, spine tumor, fracture, or neurologic disorders), 6) psychological disorders (e.g., dementia, intellectual disability, or drug abuse), 7) refusal to participate in the study, and 8) other disorders that the surgeon considers inappropriate for inclusion. This protocol adheres to the Standard Protocol Items: Recommendations for Interventional Trials (SPIRIT) guidelines for reporting of clinical trial protocols [12].

### Recruitment

Patients will be recruited if they are scheduled to undergo 1- or 2-level decompressive laminectomy for lumbar central canal stenosis in one of the six participating hospitals. Potential participants will be screened by a researcher to determine eligibility. A blinded assessor will conduct baseline assessments among the included patients. All participants will receive a baseline test and outcome assessment after giving written informed consent. No harm or adverse effects are anticipated, as outcome data will only be obtained using pre- and postoperative questionnaires and clinical and radiological assessments. Therefore, no incentives will be given to the study participants.

### Randomization

**Fig. 1:**
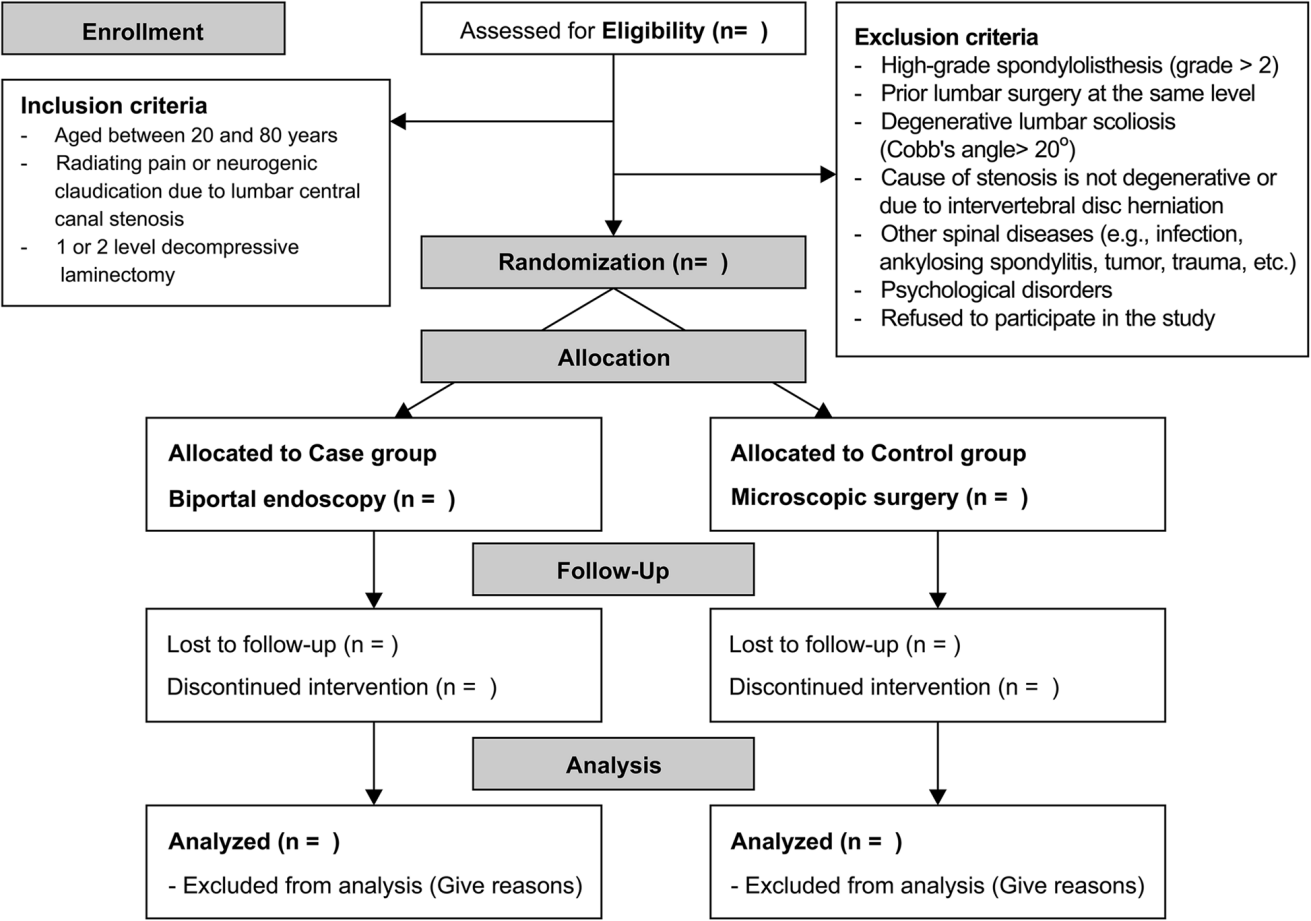
A diagram of patient flow in this study

The included patients will be randomly assigned (permuted block method) in a 1:1 ratio to either the biportal endoscopic group (case group) or conventional group (control group), following a computer-generated randomization list (Fig. 1). The randomization lists will be incorporated into a web-based electronic case report form (eCRF) platform (iCReaT; internet-based clinical research and trial, icreat.nih.go.kr) accessible to authorized researchers. To minimize bias, randomization will be performed by the research coordinator who is not involved in the treatment of the patients. Sixteen surgeons from six institutions will be informed of each patient’s group allocation via the randomization code immediately before surgery. Patients will be aware of their group allocation because of the incision site after surgery. This RCT will be single-blind (assessor-blind).

### Operation technique

#### Case group: biportal endoscopy

**Fig. 2:**
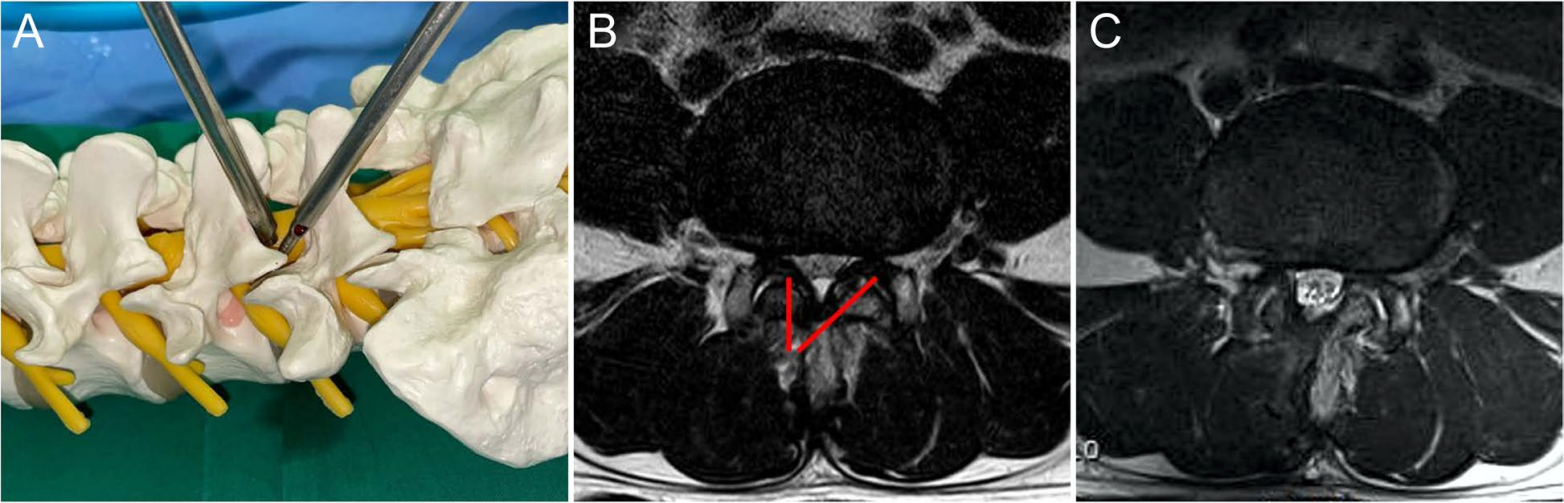
(**A**) A schematic diagram of biportal endoscopic surgery using a lumbar sawbone. (**B**) Preoperative magnetic resonance imaging. Red lines indicate the target cutting area for unilateral laminectomy bilateral decompression using the biportal endoscopic technique. (**C**) Postoperative well-decompressed state

The most critical aspect of the biportal endoscopic technique is that the procedure proceeds by securing a working space through two portals. Each portal is approximately 1 cm in size; one portal serves as the viewing portal, and the other serves as the working portal. The portals are positioned immediately lateral to the spinous process and separated by 1–1.5 cm. When operating on the patient’s left side, the viewing portal is positioned on the cranial side; the working portal is positioned on the caudal side and is within the interlaminar space. Using a Cobb elevator, the paraspinal muscle is detached from the spinous process and lamina to secure a working space. Subsequent laminectomy and flavectomy procedures are performed using the ULBD technique (Fig. 2) [5]. During the surgical procedure, continuous irrigation is performed through the viewing portal, with the pressure being adjusted to 30–40 mmHg.

#### Control group: conventional surgery

**Fig. 3:**
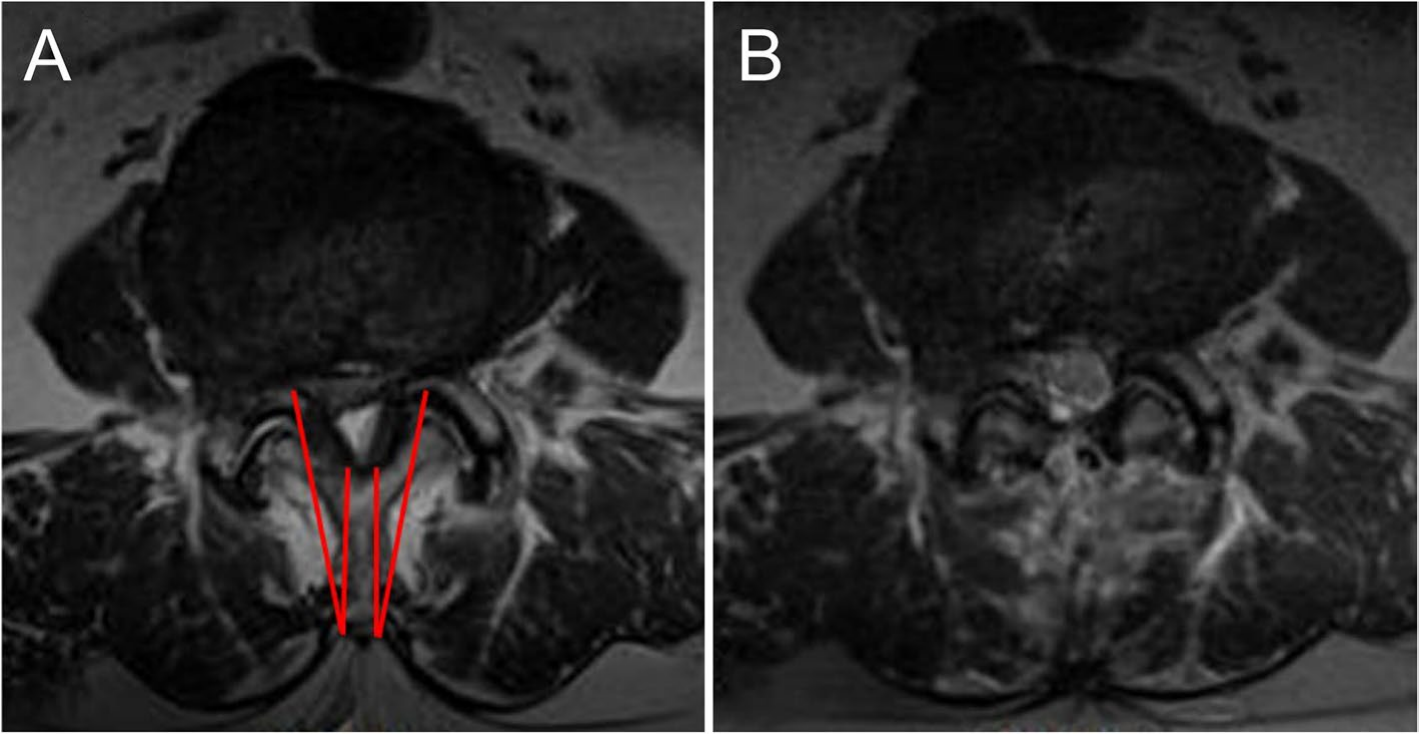
(**A**) Preoperative magnetic resonance imaging. Red lines indicate the target cutting area for conventional bilateral decompressive laminectomy performed under a microscope. (**B**) Postoperative well-decompressed state

Decompressive laminectomy has a long track record of use for the treatment of lumbar central canal stenosis [13]. A midline incision of approximately 4 cm is made, and the paraspinal muscle is detached using the Cobb elevator. After securing the operation field using a Taylor retractor, the procedure is performed under a microscope. The bilateral decompressive laminectomy procedure is subsequently performed using a burr and Kerrison punches (Fig. 3). After removing the lamina and ligamentum flavum in the region with central canal stenosis, sufficient decompression of the dura and root is confirmed.

### Outcome measures

#### Primary outcome

The primary outcome will be the difference in Oswestry Disability Index (ODI) scores between baseline and at 1 year after surgery [14]. Among the wide range of patient-reported outcomes, the ODI is the most suitable for functional evaluation of the lumbar spine. Patients are instructed to provide an answer of 0–5 for each section in the ODI questionnaire, with a score of 5 representing the greatest disability. The total score is calculated as a percentage of the total maximum score (excluding questions with no responses). In cases where a patient provides two or more responses to a single question, the highest score will be counted.

#### Secondary outcomes

##### Clinical outcomes

1. Visual analog scale (VAS) score for low back pain and radiating pain of the lower extremities: scores range from 0 to 10; a higher score indicates more severe pain
2. Patient’s quality of life, as assessed by the EuroQol 5-dimensions questionnaire (EQ-5D): EQ-5D contains five questions with five responses for each question, and the total score is converted into the final EQ-5D value, ranging from 0.000 to 1.000; higher score indicates a better quality of life [15].
3. Surgery satisfaction
4. Walking time
5. Postoperative return to daily life period
6. Surgical scars, as evaluated using the Patient and Observer Scar Assessment Scale 2.0, which contains six parameters with 10-point scoring system; 6 represents normal skin and 60 represents the worst scar imaginable
7. Surgery-related variables, such as postoperative drainage (mL), operation time (minutes), admission duration (hours), and postoperative creatine kinase (International Unit/L)

Patient-reported outcomes will be collected from the participants at baseline and at 2 weeks, 3 months, 6 months, and 12 months after surgery.

##### Radiographic outcomes

1. The degree of central canal release and canal dimension, as measured using postoperative magnetic resonance imaging (MRI) or computed tomography
2. Other radiographic complications, as assessed using simple radiographs during the follow-up period

Baseline radiographs in the anteroposterior, lateral, flexion, and extension views will be used to score spondylolisthesis and segmental instability at the surgery level. A preoperative spine MRI will be systematically checked, and the severity of the canal compromise will be evaluated based on Schizas classification [16].

Safety will be assessed by evaluating all adverse and severe adverse events; all incidents will be reported to the appropriate institution, as required. All outcomes will be recorded by the blinded assessor in the eCRF system (Table 1).

**Table 1:** Evaluation schedule

Visit type	Screening	Operation	Follow-up			
Visit	1	2	3	4	5	6
Visit week	-4–0 weeks	0–2 days	2 weeks	12 weeks	24 weeks	52 weeks
			± 5 days	± 4 weeks	± 8 weeks	± 8 weeks
Informed consent	O					
Demographics^a^	O					
Inclusion/exclusion	O					
Randomization		O				
Operation		O				
MRI (or CT)^b^	O	O				
Simple radiographs	O		O	O	O	O
ODI	O		O	O	O	O
EuroQol 5-dimensions	O		O	O	O	O
VAS	O		O	O	O	O
POSAS				O	O	O
Other surveys^c^			O	O	O	O
Adverse events		O	O	O	O	O

*MRI* magnetic resonance imaging; *CT* computed tomography; *ODI* Oswestry Disability Index; *VAS* visual analog scale for low back pain and radiating pain of the lower extremities; *POSAS* Patient and Observer Scar Assessment Scale

^a^Baseline patient characteristics, including past medical/surgical history, physical examination, and laboratory tests

^b^A CT scan will be obtained if MRI cannot be performed

^c^ Including surgery satisfaction, walking time, and postoperative return to daily life period

#### Sample size and statistical analysis

This trial will recruit 120 participants (60 participants per group) to confirm the primary outcome equivalence between biportal endoscopic and conventional decompressive laminectomy. Previous studies have indicated a minimal clinically important ODI difference of 12.8 [17], as well as a standard deviation of 18.8, at 1 year after endoscopic decompressive laminectomy [18]. A required sample size of 60 patients per group was determined based on these values and the following parameters: alpha of .05, power of .90, two-sided confidence interval of 95%, and a 20% loss to follow-up.

All statistical analyses will be performed using SPSS 26.0 (version 26.0, Chicago, IL, USA). A *P* value < .05 will be considered significant. A modified intention-to-treat (mITT) analysis will be used. The mITT strategy indicates that participants are analyzed on whether they underwent a randomly assigned surgery (to avoid the effects of crossover and dropout, which may disrupt the random assignment to the treatment groups). Participants who do not complete the follow-up schedule will be excluded from the analysis, and there will be no additional enrollment.

In each group, changes in secondary outcomes from baseline to follow-up will be analyzed with repeated measures analysis of variance. Differences in other clinical and radiologic outcomes, as well as adverse events, between the two groups will be analyzed using Student’s t-test (for continuous variables) and chi-square test (for categorical variables).

### Data management

Data from anonymized patients are entered into an electronic research database created by the government (Internet-based Clinical Research and Trial Management System). This allows investigators and researchers to safely and directly extract patient data for research purposes. Database management will be implemented through a specialized contract research company, which has extensive experience in eCRF production and management. Patient management and eCRF data input will be performed by a specialized clinical research coordinator at each of the six participating institutions. A professional clinical research associate will be responsible for auditing the clinical research data to ensure the quality and integrity of the clinical trial outcomes.

### Ethics approval

The design and protocol of this multicenter, assessor-blind, prospective RCT have been approved by the institutional review board of the six participating hospitals (Hallym University Kangnam Sacred Heart Hospital, HKS2021–02–022-002; Seoul National University Bundang Hospital, B-2102/667–003; Chung-Ang University Hospital, 2142–005 − 461; Korea University Anam Hospital, 2021AN0127; Wiltse Memorial Hospital, 2021-W02; Seoul Bumin Hospital, etc._21_004).

## Discussion

The aim of this multicenter, prospective RCT is to compare the efficacy and safety of biportal endoscopic decompressive laminectomy with those of conventional microscopic decompressive laminectomy in patients with LSS. While endoscopic spine surgery, in particular, the biportal endoscopic technique, has become an increasingly used approach in this patient group, it is not without certain challenges.

Previous studies have suggested that a sufficient level of experience is essential for performing biportal endoscopic surgery in patients with lumbar stenosis. Biportal endoscopic decompressive laminectomy has a particularly steep learning curve, as competency in this technique requires the completion of approximately 58 cases [19]. Indeed, a previous study reported that complications occurred in 7 (10.3%) of 68 cases during this learning curve [20]. Therefore, under the premise that conventional spine surgery requires skill, a learning curve of approximately 60 cases is considered necessary for the biportal endoscopic technique.

Nevertheless, previous studies have indicated that the clinical outcomes achieved with the biportal endoscopic technique are similar to those of open microscopic surgery when the learning curve is completed; the biportal endoscopic technique has also been shown to yield superior early postoperative outcomes [10, 18, 21]. According to a recent meta-analysis of 383 patients from five studies, the majority of postoperative clinical outcomes are equivalent between biportal endoscopic laminectomy and conventional microscopic laminectomy [11]. However, the biportal endoscopic technique has been shown to have additional advantages, such as less immediate postoperative back pain, a shorter length of hospital stay, earlier ambulation, a reduced need for opioids, and lower postoperative segmental instability [21–23].

To date, most of the studies on the biportal endoscopic technique have utilized a retrospective study design and have only provided preliminary results. However, the results of two recent RCTs have been reported. Kang et al. found that the use of the biportal endoscopic technique in patients with LSS resulted in more favorable clinical outcomes, such as less postoperative pain and a shorter hospital stay, compared with the use of microscopic surgery [24]. In another RCT, Park et al. reported the non-inferiority of biportal endoscopic decompressive laminectomy to microscopic open surgery in patients with symptomatic LSS [18]; therefore, they suggested that the biportal endoscopic technique was a feasible option in this patient group.

Nevertheless, there are still some doubts regarding the efficacy of biportal endoscopic surgery in patients with LSS, as the majority of studies have been retrospective and utilized short-term follow-up assessments and small sample sizes at single centers. The results reported by the two aforementioned RCTs are insufficient to conclusively determine whether there are any differences in efficacy between biportal endoscopy and conventional microscopy.

To the best of our knowledge, this study will be the first multicenter, prospective RCT to compare the outcomes between biportal endoscopic and conventional microscopic decompressive laminectomy. However, this study protocol has some limitations. First, this is not a double-blind randomized controlled trial. As mentioned previously, the participant can learn the type of surgery performed based on the skin incision. Therefore, this study is a randomized, controlled, assessor-blind clinical trial. Second, the evaluation of pain control before and after surgery may be insufficient. Third, there is heterogeneity of treatments before surgery, and there will be differences between institutions and surgeons regarding pain control regimens chosen after surgery. This may cause inaccuracies in the evaluated clinical outcomes after surgery. Despite these limitations, the results of this trial will provide a high level of evidence for the efficacy and safety of the biportal endoscopic technique in patients with LSS and facilitate the development of clinical practice guidelines. Furthermore, the results of this study may indicate the feasibility of the biportal endoscopic technique for other types of spinal surgery.

## Data Availability

The electronic database server (iCReaT) will not be publicly accessible. Access to the data set is provided only to the Data Management Committee of the Korean Government Research Consortium. The study findings will be published in a peer-reviewed journal.

## Abbreviations

LSS: Lumbar spinal stenosis
MRI: Magnetic resonance imaging
ODI: Oswestry Disability Index
RCT: Randomized controlled trial
ULBD: Unilateral laminectomy bilateral decompression
VAS: Visual analog scale
